# A cross-sectional study of the use and effectiveness of the Individual Development Plan among doctoral students

**DOI:** 10.12688/f1000research.15154.2

**Published:** 2018-07-05

**Authors:** Nathan L. Vanderford, Teresa M. Evans, L. Todd Weiss, Lindsay Bira, Jazmin Beltran-Gastelum

**Affiliations:** 1 Department of Toxicology & Cancer Biology, College of Medicine, University of Kentucky, Lexington, Kentucky, USA; 2 Markey Cancer Center, University of Kentucky, Lexington, Kentucky, USA; 3 Center for Cancer and Metabolism, University of Kentucky, Lexington, Kentucky, USA; 4 Department of Pharmacology, University of Texas Health Science Center at San Antonio, San Antonio, Texas, USA; 5 Department of Psychiatry, University of Texas Health Science Center at San Antonio, San Antonio, Texas, USA; 6 Department of Pharmacology and Toxicology, College of Pharmacy, University of Arizona, Tuscon, Arizona, USA

**Keywords:** biomedical research, career development, career planning, doctoral students, individual development plans, PhD training

## Abstract

**Background: **The Individual Development Plan (IDP) was introduced as a tool to aid in career planning for doctoral trainees. Despite the National Institutes of Health and academic institutions creating policies that mandate the use of IDPs, little information exists regarding the use and effectiveness of the career planning tool.

**Methods: **We conducted a multi-institutional, online survey to measure IDP use and effectiveness. The survey was distributed to potential respondents via social media and direct email. IDP survey questions were formatted using a five-point Likert scale (strongly agree, agree, neutral, disagree and strongly disagree). For data analysis purposes, responses were grouped into two categories (agree versus does not agree/disagree). The data were summarized as one-way frequencies and the Pearson chi-square test was used to determine the statistical significance of univariate associations between the survey variables and an outcome measure of the effectiveness of the IDP.

**Results: **Among all respondents, fifty-three percent reported that they are required to complete an IDP while thirty-three percent reported that the tool is helpful to their career development. Further, our data suggests that the IDP is most effective when doctoral students complete the tool with faculty mentors with whom they have a positive relationship. Respondents who are confident about their career plans and who take advantage of career development resources at their institution are also more likely to perceive that the IDP is useful for their career development.

**Conclusion: **Given the nuanced use and effectiveness of the IDP, we call for additional research to characterize the overall use and effectiveness of the IDP and to determine whether there are unintended negative consequences created through the use of the tool. Furthermore, we recommend an enhancement of career development infrastructure that would include mentorship training for faculty in order to provide substantially more career planning support to trainees.

## Introduction

The spotlight is bright today on the sustainability of the biomedical enterprise, especially regarding the support and general career outcomes of early career investigators and trainees ^1– 3^. There is a significant supply of PhDs and a weak market demand for faculty positions, and the majority of doctoral trainees are moving into non-faculty positions in academia, industry, government agencies, or entrepreneurship ^4, 5^. Greater career development support has been suggested by many as a key area of need to better support PhDs entering into this diverse workforce ^6^.

In 2002, the U.S. Federation of American Societies for Experimental Biology created the Individual Development Plan (IDP) as a multi-component career planning worksheet that guides doctoral trainees through a self-assessment of skills, provides a platform for the exploration of scientific career paths, aids in the development of short- and long-term career goals, and prompts the creation of action plans to achieve those goals ^7^. In 2012, *Science Careers* launched a free online version of the IDP called myIDP ^8^. In 2014, following the recommendation of the National Institutes of Health (NIH)’s Biomedical Research Workforce Working Group, the NIH implemented a policy requiring the reporting of IDP use by graduate students and postdoctoral researchers in grant progress reports ^9^. Subsequently, many academic institutions have instituted policies dictating the use of the IDP for PhD trainees. Despite these policy implementations, studies investigating the use and effectiveness of the IDP have been limited to one report that was published in 2014, which studied 233 current postdoctoral researchers, 27 former postdoctoral researchers, and 337 mentors. This study demonstrated the low use of the IDP among postdoctoral researchers (19%) and their mentors (9%), but the perceived value of the instrument was high for those who had used the tool (71% for postdoctoral researchers and 90% for mentors) ^10^. There have been recent calls to study the IDP more closely and for the NIH and other stakeholders to share the data collected on its use ^11^.

Herein, we describe the assessment of the use and effectiveness of the IDP among a sample of U.S. doctoral students. We surveyed doctoral students from at least 98 different U.S. universities in the spring and early summer of 2016 (March through June). We collected data from 663 respondents in PhD programs in the life/biological/medical (76.5%) or physical/applied sciences (23.5%), with the majority of respondents being female (70.9%) compared to their male (29.1%) counterparts ( Supplementary File 1 and Supplementary File 2). We report that approximately half (53.6%) of the respondents are required to use the IDP while about one third (33.7%) report that the tool is helpful to their career development. Further, our results suggest that the IDP is most effective when graduate students complete the tool with faculty mentors with whom they have a positive relationship. Confidence regarding career plans and use of institutional career development resources are also associated with respondents being more likely to indicate that the IDP is helpful to their career development.

## Methods

### Human subjects

This research was approved by the University of Kentucky (protocol 15-1080-P2H) and University of Texas Health San Antonio (protocol HSC20160025X) institutional review boards as a component of a health and wellbeing study. Respondents read a cover page and consented to the study by clicking the online survey web link. Subjects responded anonymously and were ensured of confidentiality.

### Survey methodology

The survey was conducted online using the secure web application REDCap. The survey was distributed to potential respondents through social media (primarily Twitter and LinkedIn) and direct email to subjects enrolled in life/biological/medical or physical/applied sciences doctoral programs across a number of different U.S. institutions ( Supplementary File 1). Eligibility criteria included being currently enrolled in a life/biological/medical or physical/applied sciences doctoral program at a U.S. institution at the time the survey was conducted. Responses were collected over a three-month period, March 2016 to June 2016. The overall study sample size was dictated by the number of respondents fitting the eligibility criteria.

### Data analysis and statistical methods

Subjects were asked to respond to the IDP questions using the five-point Likert scale strongly agree, agree, neutral, disagree and strongly disagree. For data analysis, strongly agree and agree responses were grouped together as an agree category and neutral, disagree, and strongly disagree were grouped together in a does not agree/disagree category. The survey questions relevant to this study are included as Supplementary File 4.

One-way frequencies for all respondents were calculated across all of the survey variables ( Supplementary File 2). To obtain a measure of IDP effectiveness, the Pearson chi-square test was used to assess the univariate association between all the survey variables and the outcome “I Find the IDP Process Helpful to my Career Development” only among the subset of respondents who completed an IDP (that is, those respondents who agreed with question 2 or 3 within the survey) ( Supplementary File 3). All summaries and statistical analysis were performed in SAS 9.4.

### Limitations

There are a number of limitations associated with this analysis. First, this is a cross-sectional study of a convenience sample and the results may not be generalizable. For instance, the IDP use and effectiveness rates reported herein may not be representative of those across all types of trainees within the U.S. research enterprise. As a cross-sectional study that was conducted through the use of an online survey that was deployed by email and through social media, there may be some level of subject selection bias that could lead to data and outcome bias. Additionally, since the design of the study was aimed at understanding the general use and overall effectiveness of the IDP, there may be other, perhaps more specific, nuances that may not be captured by this analysis. The data was also captured over a short period of time, and thus, respondents’ experience with the IDP outside of this timeframe may not have been captured. Some disciplines (for example, biomedical versus physical science) may also place different levels of emphasis on IDP use, and likewise, policies surrounding IDP use may vary across different disciplines. Respondents may not understand their institution’s official policies on the use of the IDP. The structure of the IDP worksheet and the procedures by which institutions enforce or recommend its use also likely vary across and perhaps even within institutions and this may influence the tools use and effectiveness even within a single institution. Lastly, the outcome measure used herein to understand the effectiveness of the IDP is subjective and is only one measure that may assess how impactful the IDP is on trainees’ career development. Future studies should analyze more defined measures of IDP outcomes including those that would allow for an understanding of the tool’s impact on academic and professional success (for example, planning that leads to research output) and career planning and decision making. Despite this study’s limitations, this is the first investigation of IDP use and effectiveness in the doctoral student population and thus this work provides a baseline understanding of the IDP in this population and it should promote additional research on the topic.

## Results

### IDP use

**Figure 1.**
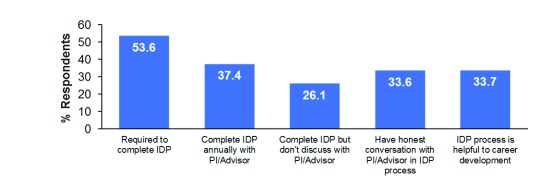
The rates of Individual Development Plan use among doctoral students. One-way frequencies for all survey respondents are shown for the variables measuring whether respondents are required to complete an IDP, complete an IDP annually with their PI/advisor, complete an IDP but do not discuss it with their PI/advisor, can have an honest conversation with the PI/advisor in context of the IDP, and whether the IDP process is helpful to their career development. One-way frequencies for all other survey variables can be found in Supplementary File 2.

Overall usage rates of the IDP among all the survey respondents was 53.6%, while 37.4% reported completing the IDP with their faculty advisor. Interestingly, 26.1% reported that they have, at some point, completed the tool but have not discussed it with their advisor. Further, 33.6% of respondents feel that they can have an honest conversation with their advisor via the IDP process and 33.7% feel that the IDP is helpful to their career development ( Figure 1 and Supplementary File 2). In the 2014 study, only 8% of postdoctoral researchers were required to complete an IDP, although overall usage among respondents was approximately 19%, and the perceived value of the tool was 71% among the postdoctoral researchers that had used the tool ^10^.

### IDP effectiveness

**Figure 2.**
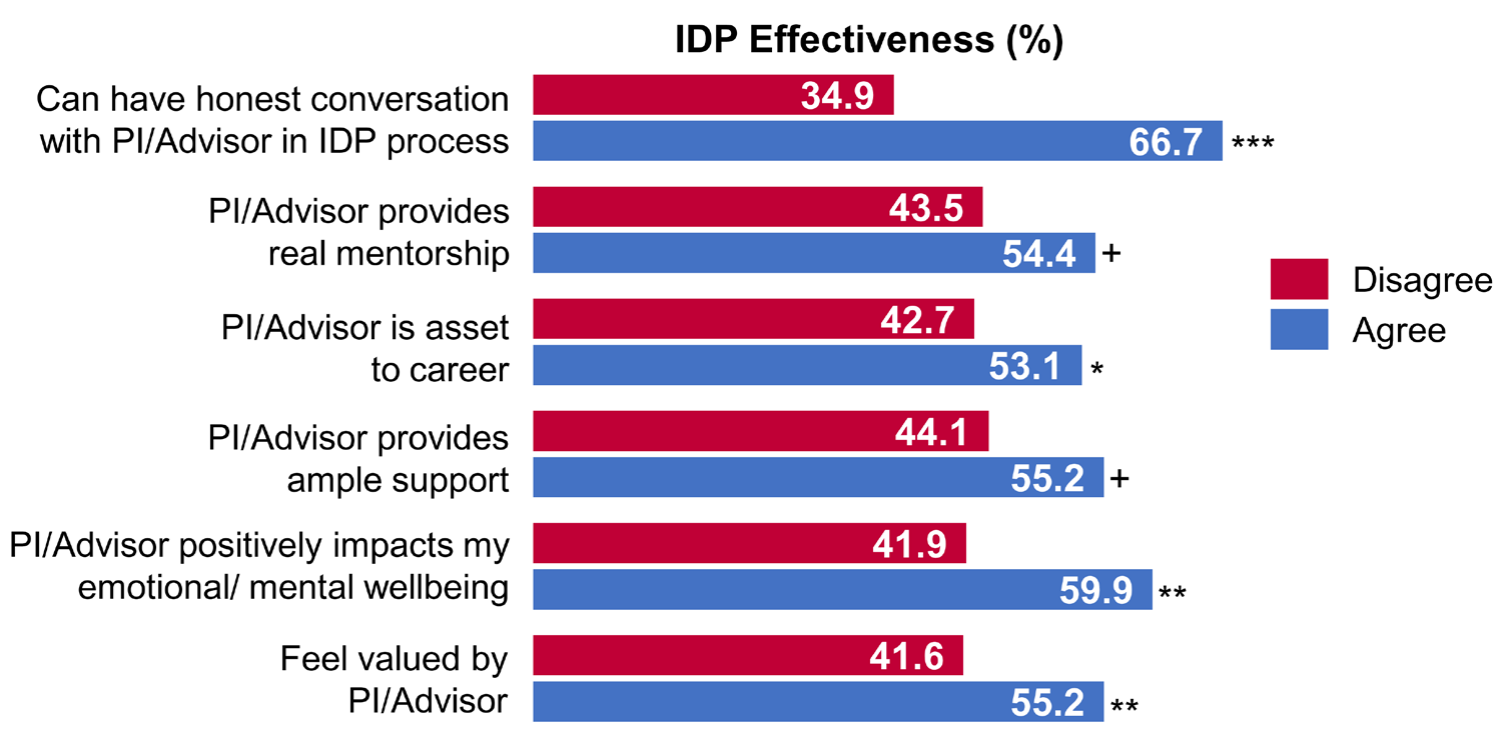
The effectiveness of the Individual Development Plan by advisor/mentor relationship. IDP effectiveness was assessed among the subset of respondents who completed an IDP by determining the univariate associations between the PI/advisor- and trainee-related survey variables and the outcome “I Find the IDP Process Helpful to my Career Development.” The Pearson chi-square test was used to measure statistical significance. *** p < 0.0001; ** p < 0.01; * p < 0.05; + p ≤ 0.07.

To gain an understanding of the effectiveness of the IDP, we analyzed the univariate association between all the survey variables and the outcome “I Find the IDP Process Helpful to my Career Development” only among the subset of respondents who completed an IDP ( Supplementary File 3). Across several measures, positive mentorship relationships associate with the effectiveness of the IDP. For example, 66.7% of those respondents who indicated that they could have an honest conversation with their PI/advisor via the IDP process versus 34.9% who could not do so found the IDP helpful to their career development (p < 0.0001). Likewise, 53.1% of those who reported that their PI/advisor is an asset to their academic and professional career versus 42.7% of those who did not agree with this statement found the IDP process helpful (p < 0.05). And, 59.9% of those who said their PI/advisor positively impacts their emotional or mental wellbeing versus 41.9% of those who did not agree (p < 0.01) found the IDP to be helpful ( Figure 2 and Supplementary File 3). These data corroborate anecdotal testimonies suggesting that supportive mentors can positively influence one’s IDP experience whereas non-supportive mentors can have the opposite impact ^12^.

Further, 57.1% of those respondents that are confident about their career prospects versus 46.3% of those who were not (p < 0.05) reported the IDP process as being helpful to their career development ( Supplementary File 3). Lastly, respondents who attend career development programs at their institution are more likely to report the IDP as helpful to their career development ( Supplementary File 3).

Individual Development Plan survey data http://dx.doi.org/10.5256/f1000research.15154.d206394
Columns Q1–Q26 correspond to the questions listed in Supplementary File 4. Click here for additional data file. Copyright: © 2018 Vanderford NL et al. 2018 Data associated with the article are available under the terms of the Creative Commons Zero "No rights reserved" data waiver (CC0 1.0 Public domain dedication).

## Discussion

More than 15 years after the creation of the IDP and 4 years after the NIH required its use, do we know if the tool is working as it was intended? Unfortunately, the answer is no. The study focusing on postdoctoral researchers from 2014 ^10^ and the current study cannot fully answer this question, but rather these studies should serve to elicit further discussion on how to best use the IDP, especially in relation to the enforcement of the tool’s use and its use with PIs/advisors. Further, this work should stimulate additional research on the general use and effectiveness of the tool.

Should policymakers, leaders of academic institutions, individual faculty, career development specialists, and even trainees find it concerning that IDP use and effectiveness is not well understood despite the tool’s general acceptance and use at countless U.S. universities and the NIH’s requirement for reporting on the use of the IDP? Should we not have known more about such an instrument prior to it being mandated as a policy? Is there potential harm being done by the mandated use of IDPs? Anecdotally, some doctoral students and postdoctoral researchers report that faculty sometimes reject non-academic career trajectories within the context of the IDP and these faculty try to force trainees toward an academic career path ^12^. Such mentorship relationships may partially explain the cause of the high rates of anxiety and depression in the doctoral student population ^13^. We believe that these questions and issues highlight the need for more work to be done in order to better understand the IDP and its effective use.

We have noticed that the structure of some IDPs has changed over time. For example, the University of Kentucky College of Medicine’s IDP has excluded the career exploration section of the tool ^14^, which was prominently included in its original design. How widespread is such a change to the IDP? Could such a change have been made to appease stakeholders who are most interested in training PhDs to pursue faculty careers? Could such a change be driving a general increase in IDP usage among faculty mentors? These questions should be addressed in future research.

Given the NIH’s adoption of the IDP, we believe that the agency should support a more extensive longitudinal study with a larger sample size to understand the barriers that are preventing some trainees and mentors from using the IDP and to better understand the effectiveness of the IDP as doctoral students and postdoctoral researchers move through their PhD education and training experience. The IDP’s impact on specific outcomes, including career path decision making and long-term career outcomes, should be studied. Future work should also determine if there are any unintended negative consequences associated with IDP use.

Career development support and related infrastructure for PhD trainees has been suggested as a being critical to sustaining the biomedical workforce ^6^. Based on our findings that positive mentorship relationships and use of career development programming are associated with a greater likelihood of trainees finding the IDP effective, we call for policymakers, funding agencies, and universities to establish and test new interventions that will support the career development of PhD trainees. For example, our data point to a need to focus attention on mentorship training for faculty and building career development infrastructure. If the NIH is to require the use of the IDP, they should require training of mentors on how to best support the career development of their mentees to obtain maximum impact, and institutional career development infrastructure is needed to achieve this. The NIH BEST program laid the foundation for building career development infrastructure at a limited number of institutions ^15^. The National Institute of General Medical Sciences has recently incorporated career development components into their pre-doctoral T32 mechanism ^16^, which is another good start to developing more widespread career development infrastructure. Other grant mechanisms should likewise be established so that a greater number of institutions can obtain NIH funds that will drive the creation of innovative career development programs across the U.S. Such programs should serve the needs of doctoral students and postdoctoral researchers and train faculty on the fine science and art of mentorship. Programmatic evaluation should be established to test the effectiveness of any interventions put into place and the results should be disseminated.

The NIH and several professional societies have been conducting “Train-the-Trainer” events to provide career and professional development training to faculty and staff. We recommend the extensive expansion of this program and evaluation of its effectiveness. The NIH could mandate such training for all faculty who pay doctoral students or postdoctoral researchers from NIH funds. Generally, it would likewise be prudent for universities to mandate that all faculty employing/supervising graduate students and postdoctoral researchers complete such training. The training could be developed and offered at each university through institutional career development offices. Studies should be developed to test whether such an intervention enhances the career development of trainees.

Ultimately, the sustainability of the biomedical enterprise hinges upon the next generation of PhDs entering the diverse workforce. We should work to support this group of scientists with sufficient career development support at the same level of rigor and reproducibility that we strive for everyday as we conduct our experiments. The IDP is likely useful for supporting the career development of PhDs, but more work is needed to understand how best to use the tool.

## Data availability

The data referenced by this article are under copyright with the following copyright statement: Copyright: © 2018 Vanderford NL et al.

Data associated with the article are available under the terms of the Creative Commons Zero "No rights reserved" data waiver (CC0 1.0 Public domain dedication). 

**Dataset 1. Individual Development Plan survey data.** Columns Q1–Q26 correspond to the questions listed in Supplementary File 4. DOI: 10.5256/f1000research.15154.d206394 ^17^.

## Supplementary material

**Supplementary File 1. Self-reported institution of all respondents.**

**Supplementary File 2. One-way frequencies of all respondents, separated by demographic characteristics.**

**Supplementary File 3. Univariate analysis of the survey’s variables and the perception of Individual Development Plan helpfulness among respondents who completed an IDP.**

**Supplementary File 4. Example copy of the survey questions relevant to this study.**
