# Supplementary material for: A cross-sectional study of the use and effectiveness of the Individual Development Plan among doctoral students
Source: F1000Res. 2018 Jul 5;7:722. Originally published 2018 Jun 11. [Version 2] doi: 10.12688/f1000research.15154.2 (PMC6039936; doi:10.12688/f1000research.15154.2)
Supplement: Supplementary file 5 [file f1000research-7-16898-s0004.tgz › eeabd3f1-c479-4230-9bf0-6f7bc847c20f.pdf]

IDP Questions

		Strongly agree	Agree	Neutral (neither agree or disagree)	Disagree	Strongly disagree
1)	My institution/college/department/PI /advisor requires me to complete a formal IDP.	○	○	○	○	○
2)	I complete an IDP annually with my PI/advisor.	○	○	○	○	○
3)	I complete an IDP but I do not discuss it with my PI/advisor.	○	○	○	○	○
4)	I find the IDP process helpful to my career development.	○	○	○	○	○
5)	I find that I can have an honest conversation with my PI/advisor via the IDP process.	○	○	○	○	○

My PI/advisor...

		Strongly agree	Agree	Neutral (neither agree or disagree)	Disagree	Strongly disagree
6)	Provides real mentorship	○	○	○	○	○
7)	Is an asset to my academic and professional career	○	○	○	○	○
8)	Provides ample support	○	○	○	○	○
9)	Positively impacts my emotional or mental wellbeing	○	○	○	○	○

I feel valued by...

		Strongly agree	Agree	Neutral (neither agree or disagree)	Disagree	Strongly disagree
10)	My mentor/advisor.	○	○	○	○	○

I am...

		Strongly agree	Agree	Neutral (neither agree or disagree)	Disagree	Strongly disagree
11)	On track to complete my training	◯	◯	◯	◯	◯
12)	Well prepared for completing my training	◯	◯	◯	◯	◯
13)	Confident about my career prospects	◯	◯	◯	◯	◯
14)	Prepared for my post-training career	◯	◯	◯	◯	◯

15) Indicate your access and thoughts toward career development programs.

- ◯ Programs are not available to me
- ◯ Programs are available, but I do not attend or attend infrequently
- ◯ Programs are available and I attend, but they have not been helpful to my current training situation nor to my future career plans
- ◯ Programs are available and I attend and they have been helpful to my current training situation and to my future career plans

Please answer the following demographic questions.

16) My ethnicity is...

- ◯ Hispanic or Latino
- ◯ NOT Hispanic or Latino
- ◯ Unknown/Not reported

17) My race is...

- ◯ American Indian/Alaska Native
- ◯ Asian
- ◯ Native Hawaiian or Other Pacific Islander
- ◯ Black or African American
- ◯ White
- ◯ More than one race
- ◯ Unknown/Not reported

18) My gender is...

- ◯ Female
- ◯ Male

19) My marital or partnership status is...

- ◯ Single
- ◯ Married
- ◯ Partnership

20) Are you a parent or a caregiver?

- ◯ Yes
- ◯ No

21) Are you a U.S. citizen or permanent resident?

- ◯ Yes
- ◯ No

22) In what country did you receive or will you receive your graduate degree?

____

23) Indicate your field of study

- ◯ Arts
- ◯ Humanities
- ◯ Social Sciences
- ◯ Business
- ◯ Law
- ◯ Engineering
- ◯ Life/Biological/Medical Sciences
- ◯ Physical or Applied Sciences
- ◯ Other

24) The degree I am seeking is...

- ◯ Doctorate
- ◯ Master's
- ◯ Other

25) I am...

- ◯ A current graduate student
- ◯ A current postdoctoral fellow
- ◯ I have completed my training and am in the workforce
- ◯ I have completed my training and am looking for a job

26) What is the name of your current academic institution?

____
