# Supplementary material for: A cross-sectional study of the use and effectiveness of the Individual Development Plan among doctoral students
Source: F1000Res. 2018 Jul 5;7:722. Originally published 2018 Jun 11. [Version 2] doi: 10.12688/f1000research.15154.2 (PMC6039936; doi:10.12688/f1000research.15154.2)
Supplement: Peer review history

# Peer review: Referee response for version 2

**DOI:** 10.5256/f1000research.16898.r35804

**Published:** 2018-07-23

**Authors:** Jessica K. Polka

**Affiliations:** 1 Whitehead Institute, Cambridge, MA, USA

Thanks for your thoughtful response. I appreciate the new analysis and written sections of the article, which greatly improve the work.

 The only reason I am not yet selecting "approved" is that I think it is important to resolve the issue Dr. Wunderlich has raised about confusion surrounding the 53% figure.

I have read this submission. I believe that I have an appropriate level of expertise to confirm that it is of an acceptable scientific standard, however I have significant reservations, as outlined above.

# Peer review: Referee response for version 2

**DOI:** 10.5256/f1000research.16898.r35803

**Published:** 2018-07-10

**Authors:** Christopher L. Pickett

**Affiliations:** 1 Rescuing Biomedical Research, Washington, DC, USA

The authors have done a commendable job in responding to reviewers' concerns. The elaboration of methods and reanalysis of data in this version satisfy the points I brought up in my initial review. This work is an important contribution to our understanding of the use of IDPs.

I have read this submission. I believe that I have an appropriate level of expertise to confirm that it is of an acceptable scientific standard.

# Peer review: Referee response for version 2

**DOI:** 10.5256/f1000research.16898.r35802

**Published:** 2018-07-10

**Authors:** Zeba Wunderlich

**Affiliations:** 1 Department of Developmental and Cell Biology, University of California, Irvine, Irvine, CA, USA

Thank you for the response to my comments. I have two minor suggestions:

1. I'm still a little confused by the percentages of IDP use. In the abstract 53% are reported to be required to complete the IDP, and the same number is reported as a usage rate in the first sentence of the results section. Does that mean that 100% of respondents required to complete an IDP did so? And 0% of non-required respondents? Also, 37.4% completed an IDP with their advisor and 26.1% didn't -- that adds up to 63.5%, not 53%. Can you explain this discrepancy?
2. At the start of the IDP effectiveness section, can you state what fraction of those that complete an IDP find it effective? It may be useful to put this in the abstract as well, as a point of comparison to the 33% of all respondents that find it useful.

I have read this submission. I believe that I have an appropriate level of expertise to confirm that it is of an acceptable scientific standard.

# Peer review: Referee response for version 1

**DOI:** 10.5256/f1000research.16508.r35352

**Published:** 2018-06-27

**Authors:** Zeba Wunderlich

**Affiliations:** 1 Department of Developmental and Cell Biology, University of California, Irvine, Irvine, CA, USA

In this article, Vanderford, et al. examine the patterns of Individual Development Plans (IDP) usage and perceptions among biology and physics graduate students at U.S. universities. Given the increasing numbers of institutions that require IDPs for graduate students, the research questions posed in this article are timely and of potentially high impact. I recommend the following changes to the article:

1. I strongly agree with the other reviewers that a key modification is to re-do the analysis, only considering those students who have experience completing an IDP, or to clarify if the study already followed this protocol.
2. How do the demographic data (gender, race, ethnicity) compare with the eligible survey population? If the data differ from the eligible population, do you expect this to affect the results? It might be appropriate to add a brief section to the beginning of the results section describing the demographics of the data and a comment to the discussion about the caveats of an opt-in survey. (I believe this is what you are hinting at in the paragraph starting "We recognize that there are limitations..." but it could be stated more explicitly.)
3. As the first reviewer, I did not always understand the denominators of the stated percentages. For example, do 37.4% of all respondents fill out an IDP with their advisor? Or 37.4% of those that are required to complete an IDP do so with their advisor? Please review the results section to clarify this and similar statements. Also, what fraction of students required to complete an IDP actually do it? And what fraction of students not required to complete an IDP do it?
4. (Minor) The word "only" is used many times throughout the results section, which colors the interpretation of the results. I'd suggest keeping the results section more neutral, while saving the "only" statements for the discussion.
5. (Minor) It might be worth noting that there is a two year gap in the 2014 postdoctoral study and the current study, so some of the increase in IDP usage may be due to an increase in usage over time.
6. (Minor) What are the p-values of the reported differences in IDP effectiveness between males/females, physics/biology students? I found these in the Supplement, but they are worth mentioning in the main text or depicting on the figures/figure legends.
7. (Minor) I appreciate the paragraph in the discussion about the changes to IDP structure. It may be worth commenting on the differences of IDP structure between institutions or individuals. My own experience with IDPs (Vincent, et al. 2015 Molecular Cell) didn't use the myIDP platform and may have influenced my perception of IDPs. I suspect I'm not alone.

I have read this submission. I believe that I have an appropriate level of expertise to confirm that it is of an acceptable scientific standard, however I have significant reservations, as outlined above.

# Response

**Published:** 2018-07-05

**Authors:** Nathan Vanderford

**Affiliations:** University of Kentucky, USA

Dear Dr. Wunderlich,

 We greatly appreciate your review, which has aided in shaping our revised article. We have responded to your major critiques/comments below.

 In response to your critique and that of the other reviewers, we have reanalyzed the IDP effectiveness data to include only those respondents that completed an IDP. We have revised the text and figures accordingly. Of note, upon this reanalysis, the differences in IDP effectiveness between fields of study and gender were no longer significant.

 Given that our study was based on an online survey that was conducted, in part, through an open call on social media, we have no way of knowing the demographic makeup of the all the potential respondents. That said, we have characterized the demographic makeup of all the actual respondents (see Supplementary File 1 and 2). We agree that there are caveats to our methods and thus we have added an extensive description of the limitations to this work within the methods section of the article.

 We apologize that there was confusion regarding the denominator used in our analysis. We have now clarified which respondents were used in the analysis. Namely, in the analysis of IDP use, one-way frequencies were calculated based on the responses from all the respondents. In the analysis of IDP effectiveness, again, we have reanalyzed our data and now present univariate associations that were calculated based on the responses from only the subset of respondents that completed an IDP. This has been more clearly stated in the revised version of the article.

 We have revised the article such that it is now written in a neural tone; we have removed such words as “only” and “minimal” in relation to describing our own findings. Future work should address what is a reasonable/acceptable level of IDP use and effectiveness.

 We appreciate your comments regarding the comparison of our data to the 2014 postdoctoral study. We have revised the text such that we now point out the study, but we do not specifically comment on how the prior data may relate to our findings. Future work should address the current use and effectiveness of the IDP in postdoctoral researchers.

 We have now included p-values in the text and in the figure legends were applicable.

 We have added comments in our limitations section regarding how differences in IDP structures could influence our data.

 In summary, we have revised our article according to your critique/comments and that of the other reviewers, and we feel that the collective reviews have significantly strengthened our work. Thank you for your time and we look forward to reading your next review.

 Sincerely,

 Nathan L. Vanderford

# Peer review: Referee response for version 1

**DOI:** 10.5256/f1000research.16508.r34887

**Published:** 2018-06-25

**Authors:** Jessica K. Polka

**Affiliations:** 1 Whitehead Institute, Cambridge, MA, USA

In “A cross-sectional study of the use and effectiveness of the Individual Development Plan among doctoral students,” the authors present a survey of graduate students conducted with the intention of determining experience with, and attitudes toward, Individual Development Plans. Given the widespread use of IDPs in the biomedical sciences, this study has the potential to provide insights that could improve the career planning process for many PhD students.

 Like reviewer 1, I am concerned that the overly-negative conclusions in this paper are influenced by the inclusion of irrelevant respondent populations. Therefore, in addition to the revisions suggested by reviewer 1, I recommend the following modifications:

1. It is perhaps unsurprising that overall usage of the IDP is low given that only 76.5% of respondents were in the life/biological/medical sciences programs. While it’s possible that the 23.5% of respondents in physical/applied sciences are in programs that require or encourage IDP completion, these students are likely unaffected by the NIH mandate mentioned in the introduction. As such, the author should not use this sample to imply that low IDP usage indicates a failure in NIH policy, as suggested by the first two sentences of the discussion.
2. Given the valid concerns that reviewer 1 has expressed, the authors should reanalyze their data to exclude respondents who have no experience with IDPs (assuming that the survey instrument did not do this already). While survey instrument did not address this question directly, the authors could at least take a subset of respondents who answered affirmatively to Q2 or Q3. If my calculations are correct, >50% of this subset finds the IDP process helpful for career development. Therefore, when the analysis is confined to students with confirmed IDP experience, the outlook for the IDP is less bleak than the tone of the paper makes it out to be.
3. The assessment of the value of the IDP should be placed into the context of overall cost of its implementation (which I suspect is extremely low). Assuming that 50% of students that complete it annual benefit from it, this is (in my opinion) a good payoff for a very small number of hours of work for mentors and students. I would be interested to read the author’s comments on cost/benefit ratio of this intervention.
4. In the discussion, the authors express concern that the IDP has been implemented without an attending study of the benefits. Later in the same section, they call for widespread career development infrastructure and “extensive” expansion of “Train-the-Trainer” events; to abide by their own logic, they should provide evidence that this change is supported by data. Again, the benefits of these interventions should be placed into the context of their cost.

I have read this submission. I believe that I have an appropriate level of expertise to confirm that it is of an acceptable scientific standard, however I have significant reservations, as outlined above.

# Response

**Published:** 2018-07-05

**Authors:** Nathan Vanderford

**Affiliations:** University of Kentucky, USA

Dear Dr. Polka,

 Thank you for your review of our work. Your comments and critique have been critical in guiding our revisions. We respond to your major points below.  

 We appreciate your thoughtful comments regarding the tone of the first version of this article. We have now revised the article in a way that neutrally presents and describes the data; our use of words such as “only” and “minimal” within the context of our findings have been removed. Additional work will need to be done to understand an appropriate and acceptable level of IDP use and effectiveness in the PhD trainee population.

 We have added a limitations subsection (within the methods section) that speaks to several points raised by all the reviewers. Within this section, we provide cautions regarding the generalizability of the data and differences between disciplines.

 We have reanalyzed the IDP effectiveness data to focus only on those respondents that completed an IDP. Of note, in relation to the previous comments about generalizability, upon reanalysis of the IDP effectiveness data, the differences between fields and gender are no longer significant and the text and figures have been revised accordingly.

 Regarding the cost/benefit ration of the IDP, this is a very interesting and important consideration, but ultimately we feel that our thoughts on this are too speculative to include in the article itself. That said, as stated at the end of the article, we do feel that the IDP can be an effective career planning tool when used “correctly.” However, we believe that more work needs to be done to assess the “correct” way to use the IDP, especially in a way that causes no harm to trainees. We believe that it is unacceptable for any trainee to be intimidated by and/or harmed through the use of the IDP, as has been suggested to occur. Ultimately, there is not enough information available on the use of the IDP to fully understand the costs, consequences, and/or benefits of its use.

 We have revised the discussion to clarify that our recommendations are based primarily on our own findings and we call for the evaluation of any new interventions that are put into place.

 In closing, we look forward to your second review in light of our revisions that are in response to your critiques and that of the other reviewers. We feel that your comments have been critical to the improvement of this work. Thank you again for your time and expertise.

 Sincerely,

 Nathan L. Vanderford

# Peer review: Referee response for version 1

**DOI:** 10.5256/f1000research.16508.r34890

**Published:** 2018-06-21

**Authors:** Christopher L. Pickett

**Affiliations:** 1 Rescuing Biomedical Research, Washington, DC, USA

The article “A cross-sectional study of the use and effectiveness of the Individual Development Plan among doctoral students” by Vanderford et al. examines the use of Individual Development Plans among biology and physics graduate students at U.S. universities. Through the use of an opt-in survey advertised on social media, the authors received over 650 responses about the usefulness of IDPs. The goal of the study was to provide information on how widespread the use of IDPs is and how effective they are in helping students explore careers.

 Understanding the efficacy of IDPs is important as this is an increasingly important piece of the career and skills development portfolio for grad students. However, I have serious reservations regarding the survey instrument and the interpretation of the resulting data.

- It is not clear from the survey instrument or the methods section that all survey respondents completed an IDP. If respondents have never completed an IDP, their responses to questions about the efficacy of the IDP in Fig. 1 would likely skew the data in an unfavorable direction. The authors should describe whether they ensured that only those who have completed an IDP took the survey.
  - If they did not use this screen, the authors should describe how data from those who haven’t completed an IDP might affect their data on IDP efficacy and take this into account when drawing conclusions.
  - If the authors did ensure that all respondents completed an IDP, this information should be clearly stated.
  - In addition, the authors should better define the denominators of the percentages reported. For example, if 33.7% respondents find the IDP helpful, is this 33.7% of those completing the survey or 33.7% of those that completed an IDP?
  - If a student completes multiple IDPs, isn't it possible the student could agree with questions 2 and 3 (complete IDP annually with advisor and complete IDP annually but don't talk to advisor)? Overlap in these populations, as is apparent in the supplemental data spreadsheet provided, could complicate the analysis of the data in Figs. 2 and 3 as it is not clear which experiences respondents may be referencing in their answers to the questions.
  - How are we to think of the values presented here in light of the study referenced that analyzed the use of IDPs among postdocs? If the data here show that more grad students fill out IDPs than postdocs, this may provide insight into how and why different populations use IDPs.
- The negative tone of the article is surprising. The authors rightly point out that the IDP is poorly studied and this survey is one of the first analyzing use by graduate students. Therefore, this study establishes the baseline for IDP use among grad students. If the authors wish to characterize the use of IDPs as low or ineffective, the authors should take time to discuss their expectations and what previous data/experiences were used to set those expectations.
  - For example, the overuse of “only” in the first paragraph of the results communicates these values fall below the authors’ expectations. Recognizing that these values will likely never reach 100%, what constitutes broad, acceptable use of an IDP?
  - Furthermore, an IDP is supposed to help students set a path for developing skills relevant for their career. The authors should discuss what “minimal effectiveness” of IDPs means in the context of the respondents being students who do not have experience understanding how their IDP relates to securing subsequent jobs.
- The interpretation of the data from the first question of the survey “My institution/college/department/PI/advisor requires me to compete a formal IDP” should be more guarded. This question asks respondents to comment on institutional or departmental policy. Respondents may disagree with the statement either because the institution does not require an IDP or because the student does not know institutional policies around IDPs. Additionally, some institutions had dozens of respondents. How consistent are responses to this and other questions when looking at respondents from the same institution?
- The authors should provide figure legends beyond the figure titles to help the reader understand the data.

I have read this submission. I believe that I have an appropriate level of expertise to state that I do not consider it to be of an acceptable scientific standard, for reasons outlined above.

# Response

**Published:** 2018-07-05

**Authors:** Nathan Vanderford

**Affiliations:** University of Kentucky, USA

Dear Dr. Pickett,

 Thank you for your review. Your critique has been helpful as we have revised the article. Below we address the major issues you raised.

 Within the text, we have clarified which respondents were analyzed in the article, thus addressing the confusion regarding the denominators used in the analysis. In the analysis of IDP use, one-way frequencies were calculated based on the responses from all the respondents. In the analysis of IDP effectiveness, we have reanalyzed our data and now present univariate associations that were calculated based on the responses from only the subset of respondents that completed an IDP. As such, our interpretation and reporting of the data has been adjusted accordingly. Of note, upon this reanalysis, the differences in IDP effectiveness between fields of study and gender were no longer significant.

 We apologize for the confusion regarding the reporting of the 33.7% of respondents that found the IDP helpful to their career development in the top section of the univariate analysis table (Supplementary File 3). We have now removed this data from the top section of Supplementary File 3, although this data can still be found in Supplementary File 2.

 We have neutralized the tone of the article in general and specifically we have removed words such as “only” and “minimal” within the context of our findings. We agree that our assessment of the expected level of IDP use and effectiveness was speculative. We also agree that as a baseline study, more work should be done to characterize an acceptable level of IDP use and effectiveness within the PhD trainee population.

 We have added an extensive limitations subsection (within the methods section) to the new version of the paper that speaks to several issues raised by the reviewers including your points about how respondents may or may not understand or be aware of their institution’s policies regarding IDP use. We also added cautions regarding variability in responses from subjects within the same institution. There are such variabilities within our dataset and it is difficult to assess the exact reasons for this, as it could be caused by, for example, general variability between respondents based on one’s individual perception of the IDP, different interpretations of the policies and procedures around IDP use, and/or the use of different IDP formats. Future work should help clarify this issue.

 We have added additional descriptions to our figure legends to help readers understand the data and our analysis.  

 In closing, we hope that you will favorably review the revised version of the article in light of our changes based on your critique as well as that of the other reviewers. We thank you again for your comments and we strongly believe that your review has been critical in strengthening this work.

 Sincerely,

 Nathan L. Vanderford
